# Serum progesterone level in luteal phase improves pregnancy rate in fresh cycles with blastocyst embryo transfer

**DOI:** 10.5935/1518-0557.20220037

**Published:** 2023

**Authors:** Juliano Brum Scheffer, Bruno Brum Scheffer, Ana Paula de Souza Aguiar, Juliana Baumgratz Franca, Autor, Daniel Mendez Lozano, Renato Fanchin

**Affiliations:** 1 IBRRA - Brazilian Institute of Assisted Reproduction, Belo Horizonte, Brazil; 2 School of Medicine, Tecnologico de Monterrey and Center for Reproductive Medicine CREASIS, San Pedro Monterrey, Mexico; 3 University Professor - Hospital Practitioner in Reproductive Medicine, France; Hopital Foch, Suresnes, France

**Keywords:** ART, progesterone, pregnancy rate, luteal phase

## Abstract

**Objective:**

To assess the association between serum level of progesterone during stimulation and in the luteal phase with pregnancy rate in a cohort of patients undergoing in vitro fertilization and embryo transfer (IVF-ET) on day 5.

**Methods:**

Retrospective Cohort Study. Patients: 62 infertile women, aged 24-42 years, undergoing ART at our center from May 2019 to May 2021. Progesterone was evaluated during ovarian stimulation on Day 2, Day 6, and Day 8 of stimulation, day of trigger (P4dhCG), and on the day of blastocyst transfer with 5 days of progesterone supplementation (P4d5+). We also calculated the difference of P4d5+ with P4dhCG. (∆P4). Then we divided the patients into two groups based on progesterone serum levels at P4d5+; <10ng/ml (Group A), ≥10ng/ml (Group B). The Student’s t-test was performed for continuous variables; Mann-Whitney’s Test and Spearman’s Test were used where appropriate for categorical variables. p<0.05 was considered statistically significant.

**Results:**

There were positive correlations between βhCG positive with P4d5+ (*p*<0.001; Rho 0.770) and ∆P4 (*p*<0.001; Rho 0.703). The pregnancy rate doubled when the serum progesterone level was ≥10ng/ml on the fifth day of progesterone supplementation compared with P4<10ng/ml (44% vs. 21%, respectively).

**Conclusions:**

The pregnancy rate was positively correlated with the serum P4 level on the fifth day of progesterone supplementation and with the difference between the serum progesterone level in the Dd5+ / dhCG. A higher pregnancy rate was observed when serum progesterone level on the fifth day of progesterone supplementation was ≥10ng/ml.

## INTRODUCTION

Current scientific research has demonstrated the importance of progesterone serum levels in the luteal phase for embryo implantation and not only embryo ploidy. The success of reproductive treatments depends on several factors, including endometrial receptivity (Casper, 2020).

Endometrial thickness assessed by ultrasound examination has been used as a marker of endometrial receptivity. Several studies have investigated other parameters of this trait of endometrial quality, such as histological and transcriptome analysis (Díaz-Gimeno *et al*., 2011; 2013). In addition to these analyses, evidence demonstrates that serum progesterone both on the day of embryo transfer and on the day of hCG application may be associated with reproductive treatment success (Yovich *et al*., 2015; Labarta & Rodríguez, 2020; Alsbjerg *et al*., 2018).

Progesterone (P4) is a steroid hormone produced by the corpus luteum shortly after ovulation in a natural menstrual cycle. It is through the regulatory effect of progesterone that the endometrium becomes secretory and receptive, in what is known as the embryo implantation window (Lessey, 2011). Peak serum progesterone levels occur during the implantation window and have been used as a marker of endometrial receptivity in natural cycles and in reproductive treatments (Harper, 1992).

In fresh IVF cycles, luteal phase support with progesterone is widely used due to inhibition of luteinizing hormone (LH) secretion. Therefore, the exogenous administration of hormones such as progesterone is fundamental and essential for reproductive success. However, there is no consensus today over the time to start supplementation, the dose of supplementation, or the duration of use of exogenous progesterone (van der Linden *et al*., 2015; Alsbjerg *et al.,* 2020; Álvarez *et al*., 2021).

The early elevation of progesterone in the follicular phase causes premature luteinization and endometrial asynchrony, affecting embryo implantation. In contrast, in the luteal phase, a progesterone level <10ng/ml indicates luteal phase deficiency leading to infertility and recurrent miscarriage (Lawrenz *et al*., 2018; Labarta *et al*., 2011; Van Vaerenbergh *et al*., 2011). Furthermore, studies suggest that high serum progesterone levels may be equally harmful to reproductive success as low progesterone levels (Kofinas *et al*., 2015; Akaeda *et al*., 2019).

The aim of this study was to evaluate the behavior of the serum level of progesterone on the day of hCG (P4dhCG), on the fifth day of progesterone supplementation (P4dD5+), as well as the difference in the level of progesterone between d5+ and dhCG (∆P4), and the correlations between serum progesterone levels and pregnancy rate in fresh IVF cycles using the antagonist protocol.

## MATERIAL AND METHODS

### Inclusion and Exclusion Criteria

This retrospective study included 62 infertile women, aged 24-42 years, undergoing routine exploration during an unstimulated cycle that preceded ART at our center, from May 2019 to May 2021. All patients met the following inclusion criteria: i) both ovaries present; ii) no current or past diseases affecting the ovaries, gonadotropin or sex steroid secretion, clearance or excretion; iii) no current hormone therapy; iv) adequate visualization of ovaries in transvaginal ultrasound scans; v) total number of small antral follicles (3-12 mm in diameter) between 1 and 32 follicles, including both ovaries; and vi) couple with a normal karyotype. Patients with uterine and severe male fator infertility were excluded. All patients signed an informed consent form before joining the study.

### Clinical and Laboratory Protocols

The patients were started on OCPs (ciproterone and ethinyl estradiol, Diane 35; Bayer Pharmaceuticals, Germany) on day 1-2 of the menses of the previous cycle and were kept on oral contraceptives for 16 to 21 days. After a wash-out period of five days (5 days counted from the last pill), we monitored their pituitary down-regulation, and patients with adequate pituitary desensitization were treated with a starting dose of recombinant FSH (Gonal-F, Follitropin alpha; Merck-Serono Pharmaceuticals, Switzerland) ranging from 225 to 300 IU and 0.25 mg/day of a gonadotropin-releasing hormone antagonist (Cetrotide, Serono Pharmaceuticals, Switzerland) introduced on day 6. Then we adjusted the FSH dose individually, according to the estradiol (E2) response and vaginal ultrasound findings. When two follicles reached ≥ 16 mm, we administered 250 mg of a recombinant human chorionic gonadotropin (Ovidrel, Merck-Serono Pharmaceuticals, Switzerland) and retrieved the oocytes 35 to 36 hours later. We routinely performed intracytoplasmic sperm Injection (ICSI) in all the fertilization procedures. Fertilization was evident when two pronuclei were spotted. The embryos were cultured until the day of transfer (blastocyst - day 5) in IVF Global® media (Life Global, Canada), supplemented with 10% synthetic serum substitute (SSS), and blastocyst-stage embryos were graded based on Gardner’s scale (Gardner *et al*., 2000).

The same embryologist performed all embryology and embryo scoring in this study. Embryo transfer (ET) was performed with the patients having a full bladder under ultrasound guidance with a trilaminar endometrium ≥7 mm. All ETs were performed with Wallace catheters (Smiths Medical Inc., Norwell, MA) at approximately 1- 2 cm less than the uterine depth identified at the prior trial transfer. Two embryos were transferred into all patients. Luteal support was started with 1200 mg/24 h micronized vaginal P4 (Utrogestan, Besins Pharmaceuticals, France) beginning the night following ovum pickup. Serum hCG was evaluated 12 days after ET, and a transvaginal ultrasound was performed at week 5 if the β-hCG was positive. Luteal support was maintained until the 10^th^ week of pregnancy.

### Hormonal Measurements and Ultrasound Scans

Measurements of estradiol (E2) pg/ml, progesterone (P4) ng/ml, βhCG mUI/ml, and AMH ng/ml serum levels were performed at the same laboratory using an IMMULITE 2000 Immunoassay System (Siemens, Berlim, Germany), while transvaginal ovarian ultrasound scans were performed by same operator. The sensitivity of the E2, P4, βhCG, and AMH assays was 20pg/mL, 0.2ng/mL, 0.4mUI/mL, and 0.1ng/mL, respectively. All intraassay and interassay variation coefficients were <10. Progesterone was evaluated during ovarian stimulation on Day 2, Day 6, Day 8 of stimulation, on the day of trigger (P4dhCG), and on the day of blastocyst transfer with 5 days of progesterone supplementation (P4d5+). We also calculated the difference of P4d5+ with P4dhCG. (∆P4). Then we divided the patients into two groups based on serum progesterone levels at P4d5+, <10ng/ml (Group A), ≥10ng/ml (Group B).

### Statistical Analysis

Descriptive parameters and patient characteristics were reported as mean SD or median (interquartile range), depending on the distribution. Student’s t-test was used for continuous variables; Mann-Whitney’s Test and Spearman’s Test were used where appropriate for categorical variables. *p*<0.05 was considered statistically significant. All participantes gave written informed consent before joining the study. The IBRRA Ethics Committee approved the study.

## RESULTS

The 62 patients included in the study were analyzed for age (35±3.6yrs), BMI (25.9±2.2 Kg/m^2^), AMH (2.5±3.2ng/ml), AFC (12.9±6.0), total dose of FSH (2225±571IU), total days of stimulation (9.9±1.7), number of MII follicles (7.8±4.2), estradiol of dhCG (2834±909 pg/ml), P4 of dhCG (0.3±0.3ng/ml), P4 of d5+ (13.7±12.2), ∆ P4 (13.3±12ng/ml), MII follicles (6.2±3.6), A+ B embryos (1.9±0.5).

More than a third of the patients (33.87%) had positive βhCG levels and 66.13% had negative levels.

The correlations between ovarian reserve markers, age, AMH, and AFC and other variables are shown in Table 1.

**Table 1:** Correlation of Age, AMH, and AFC with other variables (Spearman’s Test).

	Estradiol of dhCG (pg/ml)	FSH total dose (IU)	Days of stimulation	MII follicles	P4 dhCG ng/ml	P4 d 5+ ng/ml	∆P4 ng/ml	Embryos A+B	βhCG
Age (yrs)	<0.001	0.02	0.2	<0.001	0.4	0.1	0.1	0.08	0.5
AMH (ng/ml)	<0.0001	<0.0001	0.05	<0.0001	0.1	0.1	0.1	0.03	<0.001
AFC	<0.0001	<0.0001	0.01	<0.0001	0.2	0.01	0.01	0.004	<0.0001

* *p*<0.05 - significance

**Figure 1:**
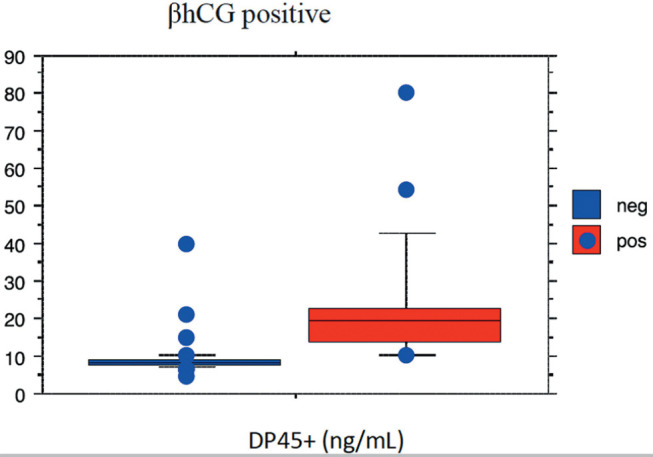
Correlation between a positive βhCG level and P4d5+ (*p*<0.001; Rho 0.770).

**Figure 2:**
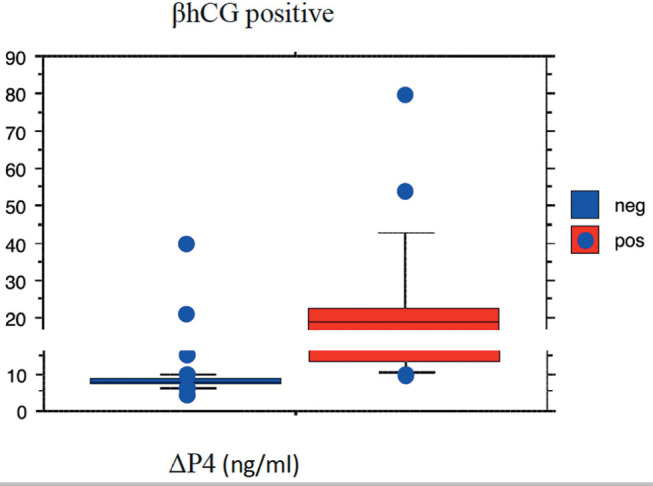
Correlation between βhCG positive with ∆P4 (*p*<0,001; Rho 0.703).

Positive correlations were found between a positive βhCG level and P4d5+ (*p*<0.001; Rho 0.770) and with ∆P4 (*p*<0.001; Rho 0.703) (Figure 1 and Figure 2).

The patients were divided into two groups based on serum P4 levels on the fifth day of progesterone supplementation. Group A included patients with P4<10ng/ml and Group B patients with P4≥10ng/ml (Table 2). The correlation between P4 and βhCG levels (*p*<0.001) is showns in Table 3.

**Table 2:** Comparison of Groups A and B (Student’s Test).

	Group A (n-34) <10ng/ml	Group B (n-28) ≥10ng/ml	*p*
Age (yrs)	35.3±4.3	34.5±2.4	0.4
AFC	11.3±5	14.7±6.7	0.02
AMH (ng/ml)	2.0±2.2	3.1±4.1	0.1
Embryos A+B	1.8±0.5	2.0±0.4	0.1

* *p*<0.05 - significance

**Table 3:** Correlation of Groups A and B with βhCG (Mann-Whitney Test) *p*<0.001.

	Pregnancy rate
Group A (n - 34) < 10ng/ml	21%
Group B (n - 28) ≥ 10ng/ml	44.2%

* *p*<0.05 - significance

## DISCUSSION

Our study evaluated the positive impact of serum luteal phase progesterone level on pregnancy rates in fresh IVF cycles in which the antagonist protocol was used. As shown, the pregnancy rate was positively correlated with the serum P4 level on the fifth day of progesterone supplementation and with the difference between the serum progesterone level on Dd5+ / dhCG. Furthermore, we observed that serum progesterone levels on the fifth day of progesterone supplementation ≥10ng/ml were correlated a pregnancy rate twice as high as the one seen in patients with P4<10ng/ml (44% *vs*. 21%, respectively).

Regarding luteal phase supplementation with progesterone, we used vaginal micronized progesterone at a dose of 1200mg/day (400mg three times daily) due to its convenience for the patient and since it is the most common mode of administration in reproductive treatment, although several dosage schemes are available in the literature (Polat *et al*., 2020; Shiba *et al*., 2019; Wang *et al*., 2015; Zarei *et al*., 2017; Dal Prato *et al*., 2008; Asoglu *et al*., 2019; Devine *et al*., 2018; von Eye Corleta *et al*., 2004; Levy *et al*., 2000).

We observed that the only ovarian reserve marker that correlated with serum progesterone level on the fifth day of supplementation was AFC. The probable explanation for this result is that the luteal phase progesterone level is a consequence of the number of corpora lutea from the follicles recruited in ovarian stimulation, thus reflecting the intensity of the ovarian response. Therefore, patients who have a greater number of recruited follicles, theoretically, have better ovarian reserve and, as a consequence, better prognoses in terms of pregnancy rate, possibly explaining the result found in this study.

Despite this interpretation, a luteal phase deficiency is observed in 31% of the women with ovulatory cycles. Recent work has observed a prevalence of 37% of women with serum progesterone levels on the day of transfer <10ng/ml despite having good ovarian reserve (Gaggiotti-Marre *et al*., 2020).

A systematic review and meta-analysis suggested that progesterone supplementation reduces the miscarriage rate in women with recurrent miscarriage (Haas *et al*., 2019). However, another study in which progesterone supplementation was administered after a positive pregnancy test did not find a reduction in the risk of miscarriage with the use of progesterone (Coomarasamy *et al*., 2015).

Furthermore, there is no consensus over the use progesterone in the luteal phase for patients undergoing reproductive treatments. Cédrin-Durnerin *et al*. (2019) demonstrated that women with a progesterone level < 10ng/ml on the day of embryo transfer had fewer live births than women with a progesterone level above 10ng/ml. However, Volovsky *et al*. (2020) did not observe any difference in pregnancy or live birth rates in women with progesterone levels <10ng/ml. Similarly, Álvarez *et al*. (2021) did not observe differences with progesterone levels <10.6ng/ml. In induced cycles, after the administration of estradiol and exogenous progesterone, studies have suggested that the serum level of progesterone for a normal endometrial histology may be below 2.5ng/ml, but the normal gene expression is around 8 to 18ng/ml (Young *et al*., 2017). Furthermore, in ovulatory cycles luteal phase progesterone levels <5ng/ml occur 8.4% of the time, while levels <10ng/ml occur 31.3% of the time, demonstrating the need to elucidate the importance of P4 levels in the luteal phase (Schliep *et al*., 2014).

Because of these considerations and since it is currently the most adopted value in scientific research, we chose 10ng/ml as the cutoff value to divide patients into two groups and look into its relationship with pregnancy rate (Jordan *et al*., 1994; Gaggiotti-Marre *et al*., 2019).

Another possible relationship between the low level of progesterone in the luteal phase and lower pregnancy rates is the possible relationship between older female age associated with anomalies in luteal phase and embryo aneuploidies (Santoro *et al*., 1996; Mersereau *et al*., 2008).

As for the luteal phase supplementation time, we use up to 10 weeks of gestation as the main medical conduct in patients undergoing reproductive treatment (Di Guardo *et al*., 2020). Based on the natural cycle, the corpus luteum is the main source of progesterone until approximately 8-9 weeks of gestation, when the placenta takes over the maintenance and supply of progesterone, even though a recent review has shown that early interruption of progesterone in the luteal phase (between 4 and 7 weeks) does not change pregnancy rates (Watters *et al*., 2020).

To avoid clinical heterogeneity and consequently bias, we used the same stimulation antagonist protocol, performed the trigger with the same medication and the same progesterone supplementation for all patients, and performed embryo transfers using embryos in the same development stage. Another important point is that all transfers were performed with a trilaminar endometrium above 7mm, thus avoiding any endometrial factor that might explain different results between patients. Progesterone levels were tested at the same time (in the morning) and in the same laboratory to avoid the confounding factor of progesterone analysis methodology.

Unfortunately, we did not look into embryo ploidy, possibly a confounding factor in this study, but we analyzed the karyotypes of all patients and found that all had normal karyotypes, which limits the chances of genetic mutations in the embryos.

This study demonstrated a correlation between serum progesterone levels in the luteal phase and pregnancy rates. Since this is a retrospective study, additional work is needed to determine and confirm this correlation.

## CONCLUSION

Pregnancy rate was positively correlated with serum P4 levels on the fifth day of progesterone supplementation and with the difference between serum progesterone levels on Dd5+ / dhCG. Serum progesterone levels on the fifth day of progesterone supplementation ≥ 10ng/ml were correlated with higher pregnancy rates.
